# Whole-Genome Profiling of a Novel Mutagenesis Technique Using Proofreading-Deficient DNA Polymerase **δ**

**DOI:** 10.1155/2012/860797

**Published:** 2012-05-22

**Authors:** Yuh Shiwa, Sanae Fukushima-Tanaka, Ken Kasahara, Takayuki Horiuchi, Hirofumi Yoshikawa

**Affiliations:** ^1^Genome Research Center, NODAI Research Institute, Tokyo University of Agriculture, 1-1-1 Sakuragaoka, Setagaya-ku, Tokyo 156-8502, Japan; ^2^Neo-Morgan Laboratory Inc., 907 Nogawa, Miyamae-ku, Kawasaki, Kanagawa 216-0001, Japan; ^3^Department of Bioscience, Tokyo University of Agriculture, 1-1-1 Sakuragaoka, Setagaya-ku, Tokyo 156-8502, Japan

## Abstract

A novel mutagenesis technique using error-prone DNA polymerase *δ* (*polδ*), the disparity mutagenesis model of evolution, has been successfully employed to generate novel microorganism strains with desired traits. However, little else is known about the spectra of mutagenic effects caused by disparity mutagenesis. We evaluated and compared the performance of the *polδ* *MKII* mutator, which expresses the proofreading-deficient and low-fidelity *polδ*, in *Saccharomyces cerevisiae* haploid strain with that of the commonly used chemical mutagen ethyl methanesulfonate (EMS). This mutator strain possesses exogenous mutant *polδ* supplied from a plasmid, tthereby leaving the genomic one intact. We measured the mutation rate achieved by each mutagen and performed high-throughput next generation sequencing to analyze the genome-wide mutation spectra produced by the 2 mutagenesis methods. The mutation frequency of the mutator was approximately 7 times higher than that of EMS. Our analysis confirmed the strong G/C to A/T transition bias of EMS, whereas we found that the mutator mainly produces transversions, giving rise to more diverse amino acid substitution patterns. Our present study demonstrated that the *polδ* *MKII* mutator is a useful and efficient method for rapid strain improvement based on *in vivo* mutagenesis.

## 1. Introduction

Random mutagenesis is a powerful tool for generating enzymes, proteins, metabolic pathways, or even entire genomes with desired or improved properties [1]. Due to the technical simplicity and applicability to almost any organism, chemical or radiation mutagenesis is frequently used for the generation of genetic variability in a microorganism. However, these methods tend to be inefficient because they can cause substantial cell damage when performed *in vivo *[2].

A novel mutagenesis technique using error-prone DNA polymerase *δ* (*pol*δ**), based on the disparity mutagenesis model of evolution [3] has been successfully employed to generate novel microorganism strains with desired traits [4–11]. In the disparity model, mutations occur preferentially on the lagging strand, due to the more complex, discontinuous DNA replication that takes place there. Computer simulation shows that the disparity model accumulates more mutations than the parity model, in which mutations occur stochastically and evenly in both strands [3]. In addition, the disparity model produces greater diversity because some offspring will have mutant DNA while some offspring will have nonmutated, wild-type DNA.

Several studies have shown that the disparity mutagenesis method often achieved more satisfactory results (i.e., higher mutation rate and quick attainment of the desired phenotype) than conventional methods such as the chemical mutagen, ethyl methanesulfonate (EMS) [5, 10], which is known to produce mainly G/C to A/T transitions [12]. However, little else is known about the spectra of mutagenic effects caused by disparity mutagenesis.

*pol*δ** is involved in the synthesis of the lagging strand of DNA [13]. Several mutants, including the proofreading-deficient *pol3-01* strain and several low-fidelity mutants, have been shown to elevate the mutation rate [14–18]. To generate the strains with the greatest mutagenicity, Neo-Morgan Laboratory (Kanagawa, Japan) has developed the plasmid YCplac33/*pol*δ*MKII*, expressing the *pol*δ** mutant allele with 2 mutations: one mutation to inactivate the proofreading activity (D321A and E323A) [15] and another mutation to decrease the fidelity of replication (L612M) [14, 17, 18].

With the recent advent of next-generation sequencing technologies, an accurate characterization of the mutant genome, relative to the parental reference strain, is now achievable. In fact, Flibotte et al. have analyzed the mutation spectra induced by various mutagens, such as EMS, ENU, and UV/TMP, in the whole genome of *Caenorhabditis elegans* [12]. Another group has also used these sequencing technologies to analyze the genetic variations between a parental and EMS-mutagenized strain of yeast [19].

In this study, we evaluate the performance of the *pol*δ*MKII *mutator, which expresses the proofreading-deficient and low-fidelity *pol*δ** in *S. cerevisiae* haploid strain, compared with the commonly used chemical mutagen EMS. This mutator strain possesses exogenous mutant *pol*δ** supplied from a plasmid, thereby leaving the genomic one intact. We measured the mutation rate of this mutator strain and found that the mutation frequency of *pol*δ*MKII* was approximately 7 times higher than that of EMS. We also performed high-throughput next generation sequencing with Illumina GAII to analyze the genome-wide mutation spectra produced by the 2 different mutagenesis methods and found that the mutator strain exhibited more pleiotropy and gave rise to more diverse amino acid substitution patterns. Our present study has demonstrated that a proofreading-deficient and low-fidelity *pol*δ*MKII* mutator is a useful and efficient method for rapid strain improvement based on *in vivo* mutagenesis. This mutator is also useful for studying the acceleration of evolution.

## 2. Materials and Methods

### 2.1. Plasmid

Plasmid YCplac33/*pol*δ*MKII* was constructed as follows: a 4.8 kb DNA fragment containing the *S. cerevisiae* BY2961 *pol3* gene, plus the UTR 1 kb upstream and 0.5 kb downstream, (*Mat*α* ura3-52, his3*-Δ*300, trp1*-Δ*901, leu2-3, 112 lys2-801, ade2-2*) was inserted into the *Sal*I-*Eco*RI site of YCplac33, and 3 amino acid substitutions, D321A, E323A, and L612M, were introduced into the *pol3 *gene using site-directed mutagenesis [20]. YCplac33 is low-copy number plasmid and is stably maintained in *S. cerevisiae* [20].

### 2.2. Mutator Mutagenesis

YCplac33/*pol*δ*MKII* vector (and YCplac33 empty vector as nonmutator control) was introduced into *S. cerevisiae* BY2961 strain cells using the LiCl method, and the transformants (mutator strains) were selected on synthetic complete (SC)-agar plates without uracil. Five mutator strains were picked and independently cultivated in 1 mL SC medium at 30°C for 24 h (about 30 generations) in order to introduce mutations into their chromosomes. To determine the mutation frequencies of the 5 mutator strains, aliquots were spread on SC-agar plates containing L-canavanine sulfate salt (0.06 mg/mL) (Sigma, St. Louis, MO, USA) to identify *CAN1* mutants, and incubated until resistant colonies were formed. The mutation frequencies were calculated as the number of drug-resistant colonies divided by the number of colonies on SC-agar plate without drug. Forward mutation rates at *CAN1* were determined by fluctuation analysis using these 5 independent cultures [21]. In order to fix mutations, another aliquot of the mutator culture was spread on SC-agar plates containing 5-fluoroorotic acid monohydrate (Wako) to obtain demutatorized cells curing from YCplac33/*pol*δ*MKII* vector. The genomic DNA was prepared from the demutatorized cells using the procedure described in the following section.

### 2.3. EMS Mutagenesis

*S. cerevisiae* BY2961 strain cells were suspended in 0.1 M phosphate-buffered saline (PBS) (pH 7.0) containing 1.5, 2.0, 2.5, or 3.0% ethyl methanesulfonate (EMS) and were incubated at 30°C for 1 h to introduce chromosomal mutations. The cells were washed 3 times with 5% sodium thiosulfate, suspended in sterilized water, and spread on SC-agar plates containing L-canavanine sulfate salt (0.06 mg/mL) (Sigma) to identify *CAN1* mutants. The mutation frequencies were calculated as described above. Another aliquot of the EMS-treated cell suspension was spread on a YPD-agar plate to isolate single clones. The genomic DNA was prepared from 5 single clones derived from the cells treated with 1.5% EMS using the procedure described in the following section.

### 2.4. Library Preparation for Illumina Sequencing

The genomic DNA from *S. cerevisiae* was extracted using the DNeasy Blood and Tissue kit (Qiagen, Valencia, CA, USA). Each sequenced sample was prepared according to the Illumina protocols. Briefly, 3 *μ*g of genomic DNA was fragmented to an average length of 200 bp by using the Covaris S2 system (Covaris, Woburn, MA, USA). The fragmented DNA was repaired, a single “A” nucleotide was ligated to the 3′ end, Illumina Index PE adapters (Illumina, San Diego, CA, USA) were ligated to the fragments, and the sample was size selected for a 300 bp product using E-Gel SizeSelect 2% (Invitrogen, Grand Island, NY, USA). The size-selected product was amplified by 18 cycles of PCR with the primers InPE1.0, InPE2.0, and the Index primer containing 6-nt barcodes (Illumina). The final product was validated using the Agilent Bioanalyzer 2100 (Agilent, Santa Clara, CA, USA).

### 2.5. Sequencing and Data Analysis

The 11 barcoded libraries (the parental strain BY2961, 5 colonies from the mutator strain, and 5 colonies from the EMS-treated strain) were used for cluster generation in several multiplexed flow cell lanes in the Illumina Genome Analyzer II system. Ninety-one cycles of multiplexed paired-end sequencing was performed, running phi X 174 genomic DNA as a control in a separate lane of the flow cell. After the sequencing reactions were complete, Illumina analysis pipeline (CASAVA 1.6.0) was used to carry out image analysis, base calling, and quality score calibration. Reads were sorted by barcode and exported in the FASTQ format. The quality of each sequencing library was assessed by evaluating the quality score chart and the nucleotide distribution plot using FASTX-Toolkit (http://hannonlab.cshl.edu/fastx_toolkit/).

Once the raw sequence data were curated, the reads of each sample were aligned to the S288c reference genome (http://www.yeastgenome.org/) using the BWA software (Ver. 0.5.1) with default parameters [22]. To avoid false positives and mutations from repetitive regions, we removed repetitive reads from the alignment files. We then used the SAMtools software (Ver. 0.1.9) [23] to produce the lists of mutations. To identify mutations that were produced by mutagenesis, we applied the following filtering criteria to the lists of mutations:

- the coverage at the mismatch positions should be at least 10;
- the variant is not present in the sequenced parental strain;
- indels meet a SNP quality threshold of 50 and substitutions meet a SNP quality threshold of 20 (SAMtools assigns SNP quality, which is the Phred-scaled probability that the consensus is identical to the reference);
- samples meet a mapping quality of 30 (SAMtools assigns Mapping quality, which is the Phred-scaled probability that the read alignment is wrong);
- the percentage of reads showing the variant allele exceeds 90%.

A variant must pass this filter to be considered a mutation. Alignments of all mutations were inspected by Integrative Genomics Viewer (IGV) [24]. The lists of mutations were then annotated using COVA (comparison of variants and functional annotation) (http://sourceforge.net/projects/cova). COVA was specifically designed to annotate the large number of identified mutants using the Genbank annotation files.

## 3. Results

### 3.1. Determination of Mutation Frequencies

In this study, we evaluated the performance of the *pol*δ*MKII *mutator, compared with that of the commonly used chemical mutagen, EMS. To assess EMS efficiency, *S. cerevisiae* BY2961 cells were treated with different concentrations of EMS. The lethality and mutation frequencies of the canavanine resistant colonies are shown in Table 1. At an EMS concentration of 1.5%, the mutation frequency was approximately 18-fold higher than that in the control (untreated) strain. Above 2.0% EMS, the survival rate decreased with no increase in mutation frequency. Based on this result, we decided to use cells treated with 1.5% EMS for whole-genome sequencing.

To assess the effectiveness of the mutator, we transformed the haploid BY2961 strain with a yeast expression plasmid, YCplac33/*pol*δ*MKII*, expressing the *pol*δ** mutant allele containing both the mutation to inactivate the proofreading activity (D321A and E323A) and the mutation to decrease the fidelity of replication (L612M). The mutator strain harboring the YCplac33/*pol*δ*MKII* plasmid will be referred to from here on as “mutator.” We determined the mutation frequency by resistance to canavanine. As summarized in Table 2, the mutation frequency of the mutator was approximately 132-fold higher than in the cells containing the empty vector. The forward mutation rate at the *CAN1 *(arginine permease) locus was calculated to be 7.9 × 10^−6^/cell division. These results show that the plasmid-generated mutated *pol*δ** protein effectively competes with the endogenous wild-type *pol*δ** protein that is produced from the chromosome, and the semidominant negative expression of mutated *pol*δ** was effective in introducing mutations. These results also demonstrate that the mutation frequency of the mutator was approximately 7 times higher than that of EMS.

### 3.2. Whole-Genome Sequencing

To analyze the genome-wide mutation spectra of the 2 different mutagenesis methods, we implemented a parallel sequencing approach with the Illumina Solexa technology (GAII instrument). We sequenced the parental haploid strain BY2961, each of the 5 clones from the mutator strains, and each of the 5 clones from the EMS-treated strains under nonselective conditions. Sequencing reads were aligned to the S288c reference genome using the BWA software [22]. To avoid false positives due to mutations from repetitive regions, reads mapped to multiple locations were discarded, and only uniquely mapped reads were used for subsequent analysis.

In the current study, the average genomic coverage ranged from 32× to 87× (Table 3). On average, 94.18% of the S288c reference genome was covered with at least 1 uniquely mapped read at each base. Subsequently, we analyzed the data for 2 kinds of mutational events: single nucleotide variants (SNVs) and small insertions and deletions (Indels). Illumina sequencing found 6,766 genetic differences between our parental strain BY2961 and the S288c. Mutations induced by these mutagens were identified by subtracting the parental mutations. Sequence-processing details can be found in Section 2.

### 3.3. The Mutation Spectra of Mutator and EMS

**Figure 1:**
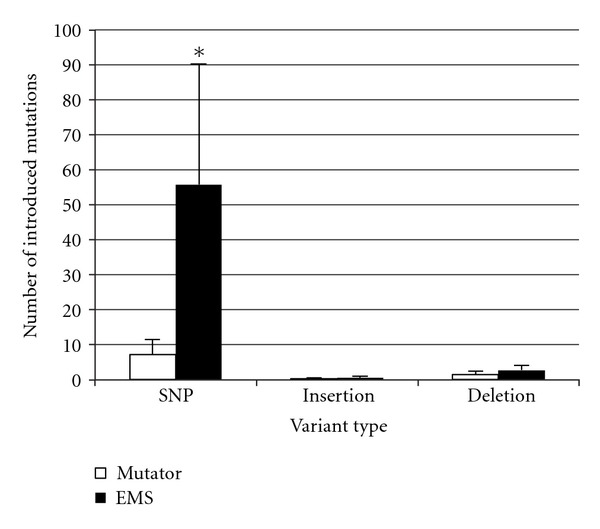
Average number of introduced mutations. By subtracting parental mutations from each mutagenized strain, we determined the number of mutations that were introduced by each mutagen. Bars represent mean ± standard error for 5 clones. *P < 0.05 versus mutator in a two-sample *t*-test.

**Figure 2:**
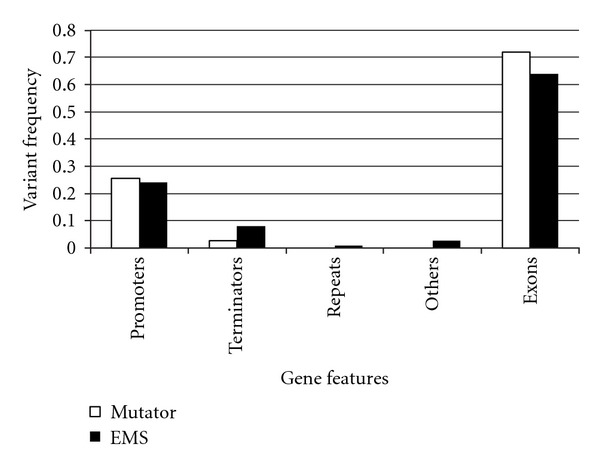
Relative frequency of SNVs affecting various gene features. The mutator and EMS generated mutations that were distributed similarly across the various gene features. The data for individual strains were combined according to the mutagen used. Promoters indicate the region 1 kb upstream of each gene. Terminators indicate the region 200 bp downstream of each gene.

We compared the average number of mutations between mutator strains and EMS-mutagenized strains (Figure 1). Mutator produced fewer SNVs than EMS (7.2 versus 55.8 per strain, resp., *P* < 0.05). Mutator and EMS produced few deletions (1.6 versus 2.8 per strain, resp.), as well as few insertions (0.2 versus 0.6 per strain, resp.). An average of 1.14 × 10^7^ nucleotide sites fulfilled our criteria of read depth (≥10), with an average base-substitutional mutation rate estimate of EMS: 4.87 (SE = 1.34) × 10^−6^ per site, Mutator: 2.09  (0.55) × 10^−8^ per site per cell division (about 30 generations). The rate we calculated for the mutator is 100-fold higher than the previously reported spontaneous mutation rate, 3.3  (0.8) × 10^−10^, based on 454 analyses of 4 mutation-accumulation (MA)-lines [26]. The 2 mutagens generate mutations that are distributed similarly across the various gene features although the mutator did produce more SNVs within exons than did EMS (Figure 2). 

**Figure 3:**
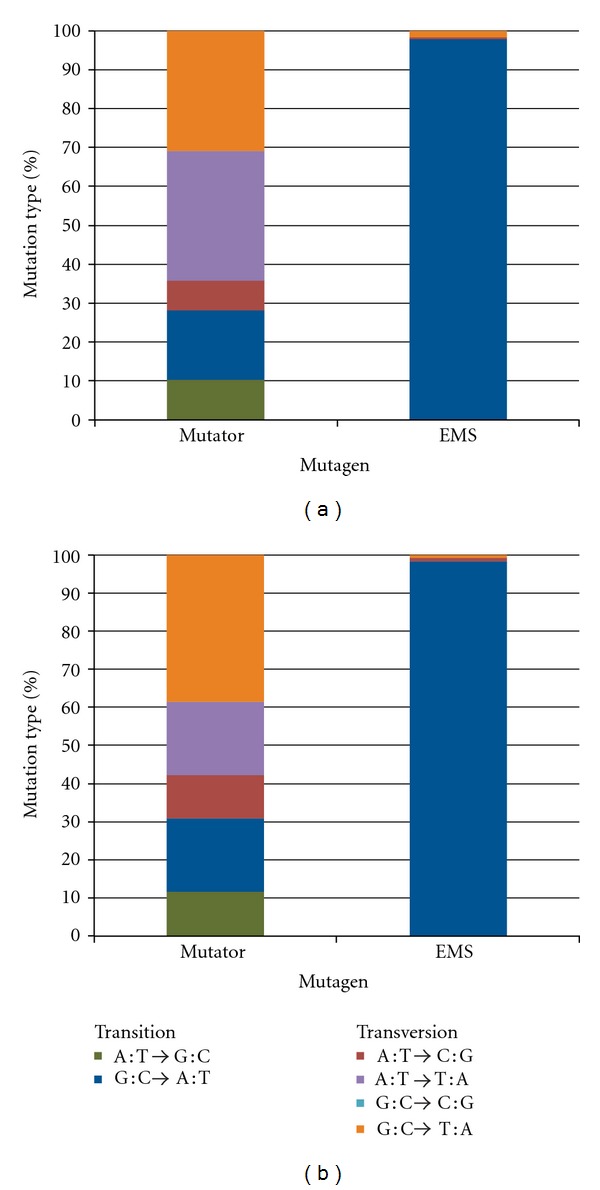
Relative frequency of transitions and transversions induced by *pol*δ** and EMS. The mutations spectra show the frequency of transitions and transversions generated by the mutator and EMS. The data for the individual strains were combined according to the mutagen used. The color key identifying the type of mutation is provided in the inset. Complementary mutations, such as A→C and T→G, are pooled. (a) Genome-wide profile; (b) Non-synonymous substitutions only.

The mutation spectra are shown in Figure 3(a). In the genome-wide profile, we found that the mutator primarily induced transversions (72%) while EMS primarily induced transitions (97%), well in accord with the known mutagenic specificity of EMS [12]. Similarly, the mutator primarily induced transversions (69%) in the nonsynonymous substitutions in exons (Figure 3(b)), similar to what has been seen in *pol3-01* study using *URA3* reporter gene [16]. EMS treatment was also in agreement with the genome-wide spectra, induced transitions with a prevalence of 98%.

### 3.4. Amino Acid Substitution Patterns

The mutation spectra of a given mutagenesis method influences the repertoire of changed amino acids at the protein level, and we were able to evaluate the amino acid substitution patterns generated by our 2 protocols (Table 4). Initially, we classified mutations into those that preserved the corresponding amino acid, changed the amino acid, or generated a stop codon. A clear difference was seen between mutator and EMS. Of the total mutations, the mutator changed the amino acid in approximately 85%, whereas EMS changed the amino acid in approximately 61%. The mutator also generated more stop codons than EMS (7% versus 2%, resp.). While mutator generated more changes to the first or second nucleotide of the codon, EMS generated changes in all 3 positions in approximately equal proportions. 

Amino acid changes were classified into conservative and nonconservative substitutions, where a conservative substitution changed the encoded amino acid to a similar amino acid according to the criteria of the BLOSUM62 matrix [25]. Of the amino acid changes, mutator produced more nonconservative substitutions than EMS (83% and 53%). For the comparison of random mutagenesis methods, Wong et al. [27] proposed a useful structure indicator that takes into account Gly and Pro substitutions as well as stop codons. In our study, the mutator produced an equivalent number of Gly/Pro and stop codon substitutions, whereas EMS generated only stop codon substitutions.

## 4. Discussion

In this study, we evaluated the performance of a novel mutagenesis technique using error-prone proofreading-deficient and low-fidelity DNA polymerase **δ** by determining the mutation rate of the strain harboring the enzyme. We also analyzed the spectra of mutations across the entire *S. cerevisiae* genome and then assessed the diversity of mutation types at the amino acid level.

Proofreading-deficient *pol*δ** mutants, such as *pol3-01* strain, and several low-fidelity *pol*δ** mutants, such as L612M, have been shown to present a mutator phenotype and to elevate the mutation rate [14–18]. We generated a BY2961 strain expressing a *pol*δ*MKII *mutator, *pol*δ** mutant allele containing a combination of mutations to inactivate the proofreading activity (D321A and E323A) and to decrease the fidelity of replication (L612M). This mutant allele acts as a strong mutator, as evidenced by the high frequency of spontaneous mutations (131-fold over control, compared to 18-fold for EMS strains). Vencatesan et al. reported the forward *CAN1 *mutation rates of *pol*δ** mutants as 1.5 × 10^−6^ in L612M, and 5.6 × 10^−6^ in *pol3-01 *[18]. These mutant strains were constructed by integrating the *pol3-01* or* pol3*-L612M allele into the chromosomal *POL3* gene by targeted integration, thereby disrupting the endogenous *POL3* gene. In contrast, our mutator plasmid expressing the *pol*δ** mutant allele produced a mutation rate of 7.9 × 10^−6^, which shows a high mutation rate as well as chromosomal integration. The use of the *pol*δ*MKII* mutator plasmid allows the continued expression of the endogenous wild-type *POL3* and provides for an efficient restoration of the wild-type mutation rate by curing the yeast strains of the mutator plasmid. Once the desired trait(s) has been selected, curing the cells from the mutator plasmid can stabilize the newly obtained phenotype.

In general, all random mutagenesis methods developed to date are biased toward transition mutations, although efforts have been made to overcome this [28]. While transition bias was observed in EMS, we actually observed transversion bias with the mutator (Figure 3(a)). Because of this, the mutator yielded a broader spectrum of nucleotide changes across the entire genome. The mutator was also biased toward transversions in the nonsynonymous substitutions (Figure 3(b)). For EMS, the spectrum of mutation events we observed is similar to what has been reported by others [12].

At the protein level, the amino acid substitution pattern differed between the mutator and EMS (Table 4). Mutations generated by the mutator resulted in amino acid substitutions more often than did mutations generated by EMS (85% versus 61%, resp.). Most of the substitutions made by the mutator were nonconservative, whereas only half of the substitutions made by EMS were nonconservative. In addition, the mutator generated more structure-disturbing amino acid changes (Gly/Pro). The transversion bias of non-synonymous substitutions by the mutator generates more diverse amino acid substitution patterns than does the transition bias of EMS.

Although the average base-substitution mutation rate of EMS was approximately 100 times higher than that of the mutator, the mutation frequency of the mutator was approximately 7 times higher than that of EMS. This gap between a higher apparent mutation frequency and fewer mutations may be explained by the higher proportion of amino acid changes and the diversity of amino acid substitutions by the mutator. This suggests one plausible explanation for the effectiveness of the disparity mutagenesis.

The disparity mutagenesis technique has been successfully applied to not only eukaryotic microorganisms such as *S. cerevisiae* [5, 7–9], *S. pombe* [9], and *Ashbya gossypii* [10], but also to prokaryotic microorganisms such as *Escherichia coli* [4] and *Bradyrhizobium japonicum* [6]. We believe that this novel mutagenesis technique has the potential to be applied to a wide variety of microorganisms.

Our present study has demonstrated that a proofreading-deficient and low-fidelity *pol*δ*MKII* mutator is a useful and efficient method for rapid strain improvement based on *in vivo* mutagenesis. It has been suggested that organisms may accelerate evolution by decreasing the fidelity of the proofreading activity of *pol*δ** in nature [29]; therefore, this mutator may also be useful for studying the acceleration of evolution.

## Figures and Tables

**Table 1:** Relationship between mutation frequency and survival after EMS treatment.

EMS concentration (%)	Mutation frequency of canavanine resistant (×10^−7^)	Fold elevation*	Survival (%)
0.0	2	1	100
1.5	35	18	51
2.0	36	19	30
2.5	33	17	21
3.0	37	19	12

* Fold elevation is relative to untreated cells.

**Table 2:** Frequency of drug-resistant mutants in the mutator strains.

Plasmids	Mutation frequency of canavanine resistant (×10^−7^)	Fold elevation*
YCplac33	3.70	1
YCplac33/*pol*δ*MKII *	486.7 ± 145.0^#^	132

*Fold elevation is relative to empty vector.

^#^Mean ± standard deviation of 3 SC plates.

**Table 3:** Sequencing and mapping statistics.

Sample name	Number of mapped unique reads	% mapped reads	% genome covered* by unique reads	Average coverage by unique reads
BY2961	11,155,487	96.13	94.97	87.9×
EMS1	5,406,681	96.94	94.81	42.2×
EMS2	6,240,554	97.26	94.85	48.7×
EMS3	5,275,583	98.12	94.81	41.2×
EMS4	4,502,271	97.17	94.80	35.2×
EMS5	4,113,345	96.27	94.83	32.1×
Mutator 1	9,612,541	93.93	94.90	75.8×
Mutator 2	5,111,531	92.39	94.79	39.9×
Mutator 3	5,649,822	96.11	94.95	44.1×
Mutator 4	4,226,405	98.79	94.85	33.0×
Mutator 5	9,855,938	97.36	95.10	77.6×

*Coverage is defined as the percentage of bases in the genome that have at least 1 uniquely mapped read at that position.

**Table 4:** Mutations at protein level.

	Mutator		EMS	
	*n*	%	*n*	%
Total mutations	28	100	201	100
Preserved amino acids	2	7.1	74	36.8
Amino acid changes	24	85.7	123	61.2
Stop	2	7.1	4	2.0
Changes in codon letter	28	100	201	100
1st	11	39.3	64	31.8
2nd	13	46.4	65	32.3
3rd	4	14.3	72	35.8
Impact of amino acid change	24	100	123	100
Conservative^a^	4	16.7	57	46.3
Nonconservative	20	83.3	66	53.7
Stop and Gly/Pro codons	4	15.4	4	3.1
Stop	2	50.0	4	100.0
Gly/Pro	2	50.0	0	0.0

^a^Conservative and nonconservative amino acid substitutions were defined according to the BLOSUM62 matrix [25].

## Abbreviations

EMS: Ethyl methanesulfonate
SNV: Single nucleotide variant
Indel: Insertions and deletions
SC: Synthetic complete.
